# Delayed PresentationofRenal Arterio-VenousFistula and Pseudoaneurysm in Native Left Kidney in Allograft Recipient: A Case Report

**DOI:** 10.31729/jnma.4494

**Published:** 2019-08-31

**Authors:** Amey Narkhede, Ajit Yadav, Manish Malik, Manish Kiran Shrestha, Arun Gupta

**Affiliations:** 1 Department of Interventional Radiology, Sir Ganga Ram Hospital, Rajinder Nagar, Delhi, India

**Keywords:** *renal arterio-venous fistula*, *renal biopsy*, *renal pseudoaneurysm*

## Abstract

Iatrogenic complications due to renal biopsy majorly include formation of an arterio-venous fistula, pseudoaneurysm or arterio-ureteral fistula. These complications are observed within a span of few days post biopsy and are rare after few years. We reported a case of32-year-old renal allograft recipient male presenting 6 years post biopsy of the left kidney with left lumbar region pain who was eventually diagnosed with arterio-venous fistula and pseudoaneurysm involving inferior interlobular branch of left renal artery. Superselective embolization was achieved using coils and high concentration glue and transient placement of a venous occlusion balloon with complete technical and clinical success.

## INTRODUCTION

The occurrence of a Renal Arterio-Venous Fistula (RAVF) with simultaneous presence of a Renal Artery Pseudoaneurysm (RAP) due to iatrogenic causes has a very low incidence rate. The most common cause has been described to be renal biopsy with higher prevalence in the biopsy of allograft kidneys as compared to the biopsy of native kidneys.^1^ If symptomatic, they present within few hours to days and delayed presentation are noted within 3 to 5 months post procedure. However, only a few case reports exist in the literature describing the delayed presentation of RAVF with RAP many years after percutaneous renal biopsy.^2^

## CASE REPORT

A 32-year-old male patient presented with chief complaints of severe abdominal pain in the left lumbar region associated with recurrent episodes of vomiting. The patient was an ABO compatible renal allograft recipient (2013). His baseline creatinine level was 4 mg/dl. The patient was admitted for further evaluation and management. He was found to have creatinine level of 6.23 mg/dl. He underwent a non-contrast CT scan which showed a suspicious pseudoaneurysm in the inferior interlobular branch of native left renal artery. The ultrasound of the left native kidney was suggestive of pseudoaneurysm with a suspicious Arterio-Venous Fistula (AVF). On retrospective analysis of the past history it was noted that the patient had undergone left renal biopsy prior to transplant in the year 2012. The patient was taken up for conventional angiography.

**Figure 1a-b.**
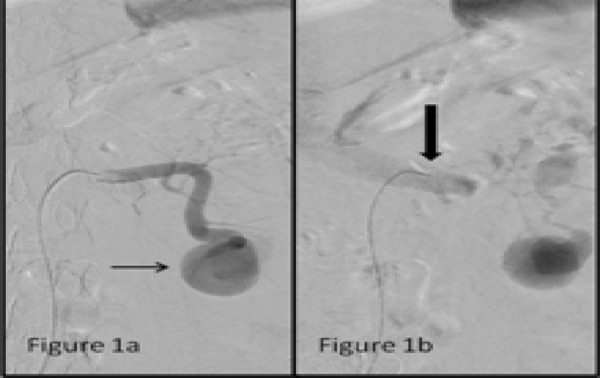
Left main renal artery angiogram shows a pseudoaneurysm (thin arrow) arising from the inferior polar branch with hypertrophy of the feeder branch.; Late arterial phase shows filling up of the left main renal vein (thick arrow) without appearance of the renal blush indicating arteriovenous fistula formation.

The Digital Subtraction Angiography (DSA) of the left main renal artery was suggestive of a pseudoaneurysm originating from the inferior interlobular branch with presence of an arteriovenous fistula (Figure 1a, 1b).

**Figure 2a-b.**
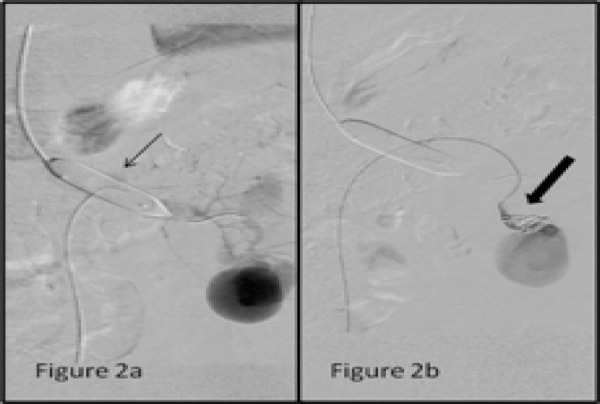
Tip of diagnostic catheter in the left main renal artery with contrast injection. Stasis of contrast is noted in the pseudoaneurysm and its outflow.; Tip of diagnostic catheter at the afferent artery to the pseudoaneurysm. Contrast injection shows persistent filling of the contrast even after deployment of coils (straight arrow) at the neck of pseudoaneurysm.

Since it was a high flow vascular malformation we introduced a balloon of 10 × 40 mm via right jugular access through a 7 Fr Sheath into the left main renal vein lumen. The balloon occluded angiogram showed decreased flow of contrast through the fistula into the main renal veinwith the balloon in inflated state (Figure 2a, 2b).

**Figure 3a-b.**
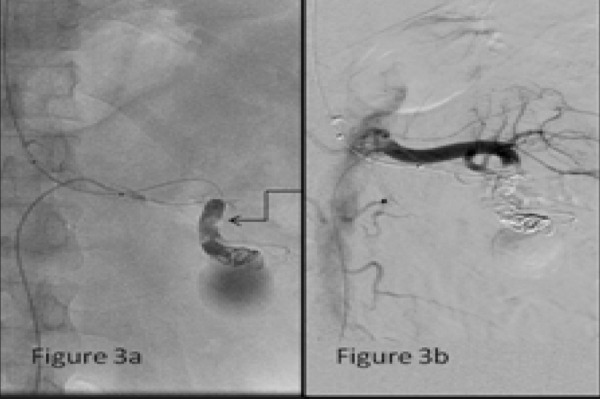
DSA image showing glue cast (elbow arrow) at the neck of pseudoaneurysm with stasis of contrast in the sac.; Angiogram through the diagnostic catheter with its tip in left main renal artery showing complete embolization of the afferent artery to pseudoaneurysm with non-filling of the pseudoaneurysm and the arteriovenous fistula.

A microcatheter was approached just proximal to the aneurysmal neck and three 8 × 80 mm MReye Embolization Coils (Cook, Bloomington, In, USA) were deployed there using scaffolding technique. Post coiling check angiogram showed incomplete occlusion with filling up of the aneurysmal sac (Figure 3a). So glue embolization using 50 % nBCA (n Butyl Cyano-Acrylate) glue was used to achieve complete occlusion of the proximal arterywith simultaneous balloon occlusion in the main renal vein (Figure 3a and 3b). Post embolization check angiogram showed non filling of the aneurysmal sac and the AVF.

The patient was stable after the procedure with gradual relief of pain. His urine output was normal and the serum creatinine level was 5.1 mg/dl. On day 2 post procedure, review ultrasound showed no evidence of pseudoaneurysm and AVF. The patient was discharged in stable condition.

## DISCUSSION

The renal AVFs are usually clinically occult in the post biopsy period with more than 95% resolving spontaneously within two years. The symptomatic RAVF having an incidence rate of 0.3–4 % and present acutely in post procedure period requiring urgent intervention. The renal pseudoaneurysms have been found to co-exist with RAVFs.^3^ The RAPs have a reported incidence rate between 1-5% post biopsy, in various studies, most of them being asymptomatic and resolving spontaneously.^4^ Both these pathologies most frequently present within a few hours to days when symptomatic. Even a delayed presentation weeks to months after the procedure is not uncommon. Review of literature, however, shows that there are very few case reports which describe symptomatic combined presentation of RAVF and RAP after a gap of years post biopsy.^2^ In our case we found a delayed presentation after 6 years. Prospective diagnosis of asymptomatic RAVF and RAP occurring beyond 3 months after the procedure is a difficult task since it is quite rare.^5^

It has been observed in various studies that the most common cause of RAVF is iatrogenic accounting for about 70% of the total RAVFs with the highest incidence being attributed to percutaneous renal biopsies with the incidence ranging from 7.4% to 11%.^6^ A difference has been observed in the occurrence of RAVF between allograft kidney and native kidney after biopsy with more frequent incidence in the allograft kidney like many other post-biopsy complications (1016% in allograft kidney vs 0.3-5-6% in native kidney).^1^ The symptomatic RAVFs can present with hematuria (micro/macroscopic), palpable thrill, abdominal bruit, severe hypertension, deterioration of renal functions and high output cardiac failure. The life threatening RAVFs, however, are noted in less than 0.5% of the cases.^7^ In contrast to this the symptoms of a pseudoaneurysm are quite non-specific which makes its clinical diagnosis difficult. Ultrasound Doppler plays an important role in immediate diagnosis of RAVF/RAP even at bedside but the main stay of diagnosis still depends on CT renal angiography findings.

The choice of management currently is the superselective embolization of RAP and RAVF even in emergency settings and complex lesions.^8^ In a high flow large vascular malformation such as our case, high concentration of glue (>40%) with coils is the preferred choice of embolizing agent to achieve complete hemostasis.^4^ Also the high flow can lead to non-target embolization. This complication can be prevented by various techniques to reduce the flow rate such as placement of occlusion balloon in arterial inflow or venous outflow or both and use of double microcatheter technique.^9,10^ We achieved stasis by using a of 10 × 40 mm balloon in the left main renal vein (single venous outflow) which helped us achieve complete occlusion of the feeding artery.

In conclusion, renal arteriovenous fistula (RAVF) and renal artery pseudoaneurysm (RAP) occurring simultaneously as a delayed presentation years after undergoing a renal biopsy has been observed in a very few patients. Reaching to a diagnosis in such a case requires a careful scrutiny of the history of the patients who are symptomatic followed by prompt superselective embolization which is a proven safe and effective method of management.

## Consent:

**JNMA Case Report Consent Form**was signed by the patient and the original is attached with the patient's chart.

## Conflict of Interest:

**None.**
